# Lifestyle changes associated with participation in colorectal cancer screening: Prospective data from the English Longitudinal Study of Ageing

**DOI:** 10.1177/0969141318803973

**Published:** 2018-10-18

**Authors:** Claire Stevens, Samuel G Smith, Charlotte Vrinten, Jo Waller, Rebecca J Beeken

**Affiliations:** 1 Department of Behavioural Science and Health, University College London, London, UK; 2 Leeds Institute of Health Sciences, University of Leeds, Leeds, UK

**Keywords:** Cancer screening, teachable moment, smoking, alcohol, physical activity, diet, colorectal cancer, cohort

## Abstract

**Objectives:**

Population-based cancer screening has been described as a teachable moment for behaviour change. This research examined the effect of faecal occult blood testing (FOBT) participation on smoking, alcohol consumption, fruit and vegetable consumption and physical activity.

**Setting:**

Data were from screening-naïve men within the English Longitudinal Study of Ageing, receiving their first FOBT invitation (n = 774). Four waves of data were included in analyses (wave 4, 2008/2009 – wave 7, 2014/2015). Baseline data were from the wave prior to FOBT invitation, and follow-up data were from the next consecutive wave (two years later).

**Methods:**

The effects of FOBT participation, time and group-by-time interactions on health behaviours were investigated using generalised estimating equations. Almost two-thirds of the sample (62.5%; n = 484) had participated in FOBT.

**Results:**

Screening participants were less likely to smoke (odds ratio (OR): 0.45, 95% confidence interval (CI): 0.29–0.68) and more likely to meet fruit and vegetable consumption guidelines (OR: 1.70, 95% CI: 1.14–2.55). Smoking decreased over time (OR: 0.74, 95% CI: 0.62–0.89), but adherence to alcohol guidelines also decreased (OR: 0.71, 95% CI: 0.53–0.91). A group-by-time interaction was found for vigorous physical activity; the odds of taking part in vigorous physical activity increased for FOBT participants, but decreased for non-participants (OR: 1.40, 95% CI: 1.01–1.95).

**Conclusions:**

This research provides tentative support for FOBT as a teachable moment for increasing vigorous physical activity. However, overall, there was limited evidence for spontaneous improvement in multiple health behaviours following participation.

## Introduction

Population cancer screening has been described as a ‘teachable moment’ for health behaviour change.^1^ The term is used to describe opportunities to facilitate behaviour change, and situations where behaviour change may occur spontaneously.^2^ Research has investigated whether, without intervention, participation in cancer screening prompts positive changes to cancer-related health behaviours. One study, set within a UK flexible sigmoidoscopy trial, showed rates of physical activity (PA) and fruit and vegetable intake increased, and smoking rates decreased following participation.^3^

There is a concern that participation in cancer screening may have a negative impact on health behaviour change, by means of a ‘Health Certificate Effect’.^4,5^ In one colorectal cancer screening trial, improvements in health behaviours were observed across the sample, but it was the unscreened population that made greater improvements for smoking, PA and fruit and vegetable consumption.^6,7^ If this is the case, the unintentional effects of cancer screening participation on lifestyle may have an impact on the cancer prevention efforts and mortality reduction of cancer screening programmes.^8^ One systematic review found little evidence that negative screening results provide false reassurance to participants, although the included studies were limited in both number and quality.^9^ Two systematic reviews have concluded that there is also little support for the idea that spontaneous positive behaviour change can occur following cancer screening participation.^10,11^ The literature is limited to studies exploring the effect of lung screening on smoking,^10^ with very few studies observing multiple health behaviours within the context of other cancer screening modalities.^11^ Additionally, few studies have compared behavioural changes among screening attenders with those of non-attenders.^12^ Studies that have included a control group have generally involved samples participating in cancer screening trials, who may be more health conscious and motivated to change their behaviour compared with population screening programme participants.^6,13^

Within existing cancer screening programmes, previous research has not distinguished between participants with and without prior experience of cancer screening. In the context of cardiovascular screening, first participation in health screening is most effective at prompting behaviour change.^14^

Prior to the recent implementation of flexible sigmoidoscopy screening,^15^ men received their first invitation to an National Health Service (NHS) cancer screening programme (faecal occult blood testing; FOBT) at the age of 60. Therefore, men invited to participate in FOBT are a distinct group, and offer the least biased sample to investigate the topic of teachable moments in a cancer screening context. This study aimed to investigate whether first participation in the English NHS FOBT cancer screening programme is associated with spontaneous lifestyle changes among attenders, compared with non-attenders, in a screening-naïve population of men from an English prospective cohort study.

## Methods

Data were taken from the English Longitudinal Study of Ageing (ELSA), a biennial prospective cohort study of English adults aged over 50. The cohort was originally sampled from the Health Survey for England, with refreshment samples recruited to maintain adequate sample size and representativeness.^16^ Data were collected using computer-assisted personal interviews and self-complete questionnaires. The most recent data (wave 7) were collected in 2014–2015.

Men approaching the age of their first invitation to participate in FOBT were included in analyses. FOBT is offered biennially, via a postal home-test kit, to men and women aged 60–74, who receive their first invitation to participate shortly after their 60th birthday. Prior to the postal home-test kit, participants receive an invitation letter with a leaflet explaining the test. The leaflet highlights weight, lack of exercise and dietary factors as bowel cancer risk factors. The home-test kit involves providing three sets of stool samples over a 10-day period. Participants’ baseline data (prior to first invitation) were taken from the wave at which they were aged 57–59. Three waves of data were used to identify baseline groups: wave 4 (2008–2009; n = 210), wave 5 (2010–2011; n = 280) and wave 6 (2012–2013; n = 284; total n = 774). Wave 4 included fewer participants, as questions relating to FOBT were included part-way through data collection. The next consecutive wave of data for each participant (waves 5, 6 and 7, respectively) provided follow-up data (following first FOBT invitation). At follow-up, participants were aged 60–61. Participants outside this age range were excluded to ensure that they had been invited to participate in FOBT once only. Participants who reported a diagnosis of cancer at either time-point were excluded.

Participation in FOBT was defined as answering ‘yes’ to the question ‘Have you ever completed the NHS bowel cancer screening test using the home test kit?’ Data for ethnicity, education and occupation were taken from a person’s baseline wave. Ethnicity was categorised into white and non-white. Based on the highest level of educational qualification achieved, education was categorised into no formal qualifications, qualifications below degree level and education at degree level or above. Occupation was categorised into managerial or professional, intermediate, routine or manual and other. Baseline and follow-up data were used for demographic variables likely to change over time. For retirement status, participants were categorised as retired or not retired at each time-point. Participants were asked whether they had a long-standing illness (yes/no) and whether it was life-limiting (yes/no): used to categorise participants as having a long-standing illness which was life-limiting or not.

Participants were categorised as current smokers if they answered ‘yes’ to the question ‘Do you smoke at all nowadays?’ Participants were asked to record the number of measures of (1) spirits, (2) glasses of wine and (3) pints of beer, lager, or cider they had consumed in the past week. Based on NHS guidelines for alcohol consumption, participants were categorised as meeting guidelines for alcohol consumption if they had consumed 14 or fewer alcoholic units in the past week.^17^ From wave 5 onwards, two items assessed fruit and vegetable consumption: ‘How many portions of vegetables – excluding potatoes – do you eat on a typical day?’ and ‘How many portions of fruit – of any kind – do you eat on a typical day?’. Responses were combined to create a composite measure of fruit and vegetable consumption. Participants who consumed five or more portions each day were categorised as meeting UK guidelines.^18^ Different, non-comparable items were used prior to wave 5, meaning analyses of this variable used a reduced sample. Levels of moderate physical activity (MPA) and vigorous physical activity (VPA) were assessed using two variants of the same item: ‘Do you take part in any sports that are (vigorous/moderately energetic)’ with response options of ‘more than once a week’, ’once a week’, ‘one to three times a month’, ‘hardly ever or never’. The response options ‘more than once a week’ and ‘once a week’ were combined to determine the proportion of people participating in VPA and MPA once or more per week. UK PA guidelines advise adults should participate in at least 150 min of MPA or 75 min of VPA per week.^19^

Data were described using means and proportions. Multivariate logistic regression was used to determine demographic predictors of FOBT participation. To investigate the effect of FOBT participation on the lifestyle factors, five separate generalised estimating equations (GEE) were used. GEE is a method used to analyse longitudinal data allowing for the estimation of differences between groups (FOBT participants vs. non-participants) for an outcome, changes to an outcome over time and group-by-time interactions.^20^ Each GEE model included two main effects (group, time) and an interaction effect (group×time) and was adjusted for ethnicity, occupation, education, limiting long-standing illness, retirement status and baseline wave. The main effect for time shows whether, across the whole sample, lifestyle factors changed between baseline and follow-up. The main effect for group shows whether, across both time points, there are any differences between groups (FOBT participants/FOBT non-participants). The interaction effect assesses whether FOBT participants changed their behaviour to a greater or lesser degree than FOBT non-participants. Proportions reported alongside GEE analyses are adjusted for all demographic covariates. Statistical analyses were carried out in Stata SE 14. Previous research using this cohort and methodology has investigated lifestyle changes following a cancer diagnosis.^21^

## Results

Of the sample (n = 774), 62.5% (n = 484) reported participating in FOBT at follow-up, 95.1% (n = 736) were white and 27.7% (n = 213) were educated to degree level or above (Table 1). Among those who were employed, 46.7% (n = 345) worked in managerial and professional occupations. Percentage of participants reporting having a life-limiting long-standing illness were 24.3% (n = 184) at baseline and 24.4% (n = 189) at follow-up. Retirement increased from 12.5% (n = 95) at baseline to 24.3% (n = 188) at follow-up. Multivariate logistic regression including baseline and follow-up demographic characteristics revealed that retirement status at follow-up positively predicted FOBT participation (odds ratio (OR): 1.99, 95% confidence interval (CI): 1.25–3.15). No other demographic factors were associated with FOBT participation.

**Table 1:** Demographic characteristics of the total sample, FOBT participants and non-participants, with multivariate analyses to identify demographic predictors of FOBT participation.

	Total sample % (n)	FOBT participants % (n)	FOBT non-participants % (n)	Adjusted odds ratio (95% CI)
Ethnicity (n=774)				
White	95.1 (736)	94.0 (460)	95.2 (276)	REF
Non-white	4.9 (38)	5.0 (24)	4.8 (14)	1.17 (0.57–2.43)
Baseline education (n=770)				
Degree level or above	27.7 (213)	30.9 (149)	22.2 (64)	REF
Qualifications below degree	58.4 (450)	56.4 (272)	61.8 (178)	0.77 (0.51–1.16)
No formal qualifications	13.9 (107)	12.7 (61)	16.0 (46)	0.75 (0.42–1.35)
Baseline occupation (n=739)				
Managerial and professional	46.7 (345)	49.8 (232)	41.4 (113)	REF
Intermediate	21.2 (157)	21.2 (99)	21.3 (58)	1.02 (0.66–1.58)
Routine and manual	31.8 (235)	28.8 (134)	37.0 (101)	0.82 (0.55–1.24)
Other	0.3 (2)	0.2 (1)	0.4 (1)	0.57 (0.34–9.36)
Baseline long-standing illness (n=756)				
No	75.7 (572)	76.7 (26.18)	73.8 (203)	REF
Yes	24.3 (184)	23.3 (112)	26.2 (72)	1.03 (0.65–1.65)
Follow-up long-standing illness (n=774)				
No	75.6 (585)	77. 9 (377)	71.7 (208)	REF
Yes	24.4 (189)	22.1 (107)	28.3 (82)	0.80 (0.50–1.29)
Baseline retirement (n=761)				
Not retired	87.5 (666)	87.3 (418)	87.9 (248)	REF
Retired	12.5 (95)	12.7 (61)	12.1 (34)	0.65 (0.37–1.15)
Follow-up retirement (n=774)				
Not retired	75.7 (586)	71.9 (348)	82.1 (238)	REF
Retired	24.3 (188)	28.1 (138)	17.9 (52)	1.99 (1.25–3.15)

**Figure 1.**
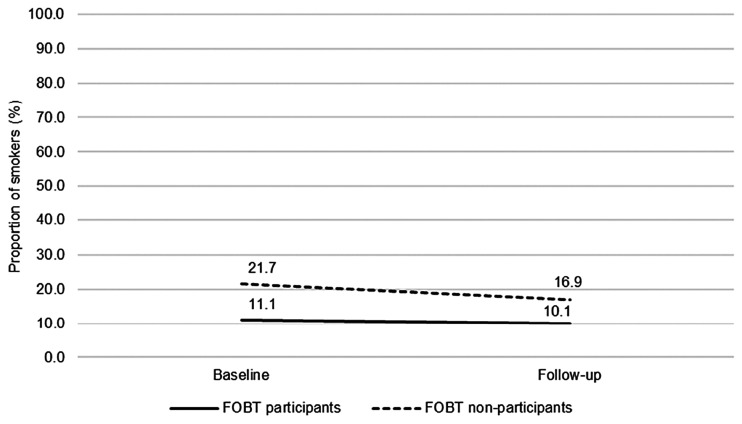
The proportion of smokers over time, comparing FOBT participants and FOBT non-participants (n = 736; FOBT participants n = 465, FOBT non-participants n = 271). Proportions adjusted for ethnicity, occupation, education, limiting long-standing illness, retirement status and baseline wave. FOBT: faecal occult blood testing.

**Figure 2.**
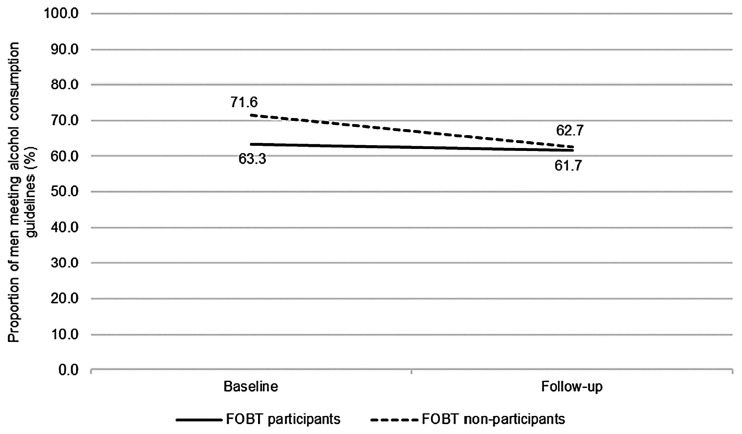
Proportion of men meeting guidelines for alcohol consumption over time, comparing FOBT participants and FOBT non-participants (n = 714; FOBT participants n = 457, FOBT non-participants n = 257). Proportions adjusted for ethnicity, occupation, education, limiting long-standing illness, retirement status and baseline wave (missing data due to participant non-response). FOBT: faecal occult blood testing.

**Figure 3.**
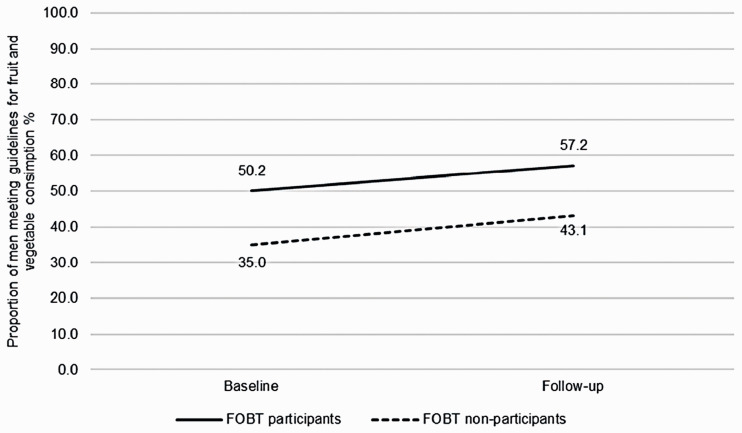
Proportion of men meeting guidelines for fruit and vegetable consumption over time, comparing FOBT participants and FOBT non-participants (n = 524; FOBT participants n = 347, FOBT non-participants n = 177). Proportions adjusted for ethnicity, occupation, education, limiting long-standing illness, retirement status and baseline wave. This analysis includes a smaller sample size due to different, non-comparable items assessing fruit and vegetable consumption prior to wave 5. FOBT: faecal occult blood testing.

**Figure 4.**
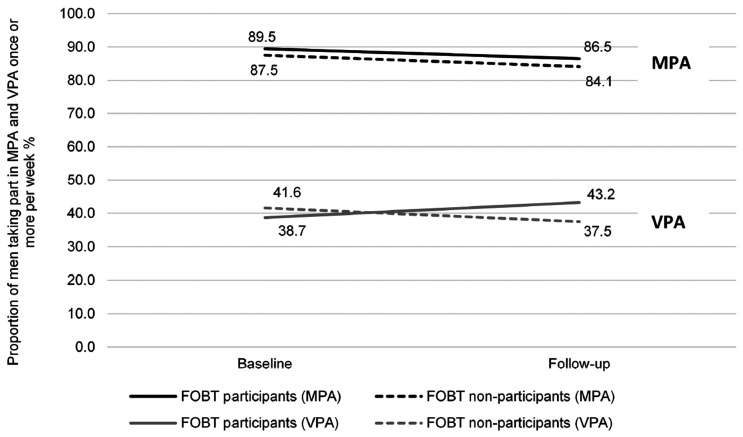
Proportion of men taking part in MPA (n = 736; FOBT participants n = 465, FOBT non-participants n = 271) and VPA (n = 734; FOBT participants n = 464, FOBT non-participants n = 270) once or more per week, over time, comparing FOBT participants and FOBT non-participants (proportions adjusted for ethnicity, occupation, education, limiting long-standing illness, retirement status, and baseline wave). FOBT: faecal occult blood testing; MPA: moderate physical activity; VPA: vigorous physical activity.

- *Smoking* (n = 736): The proportion of current smokers decreased over time (OR: 0.74, 95% CI: 0.62–0.89) from 14.9% to 12.7% (Figure 1). Fewer FOBT participants identified as smokers compared with FOBT non-participants (OR: 0.45, 95% CI: 0.29–0.68). No group-by-time interaction was observed for smoking behaviour (OR: 1.15, 95% CI: 0.90–1.47), indicating that men who participated in FOBT did not change their behaviour any more or less than non-participants.
- *Alcohol consumption* (n = 714): The proportion of men meeting current alcohol consumption guidelines decreased over time (Figure 2), from 65.9% to 61.6% (OR: 0.69, 95% CI: 0.53–0.91). There was no difference in adherence to alcohol guidelines between the screened and non-screened groups (OR: 0.87, 95% CI: 0.62–1.23). No group-by-time interaction was observed for alcohol consumption (OR: 1.34, 95% CI: 0.96–1.85). Compared with FOBT non-participants, FOBT participants were no more or less likely to change their alcohol consumption over time.
- *Fruit and vegetable consumption* (n = 524): The proportion of participants meeting guidelines for fruit and vegetable consumption did not change over time (45.0% vs. 52.7%; OR: 1.32, 95% CI: 0.91–1.90) (Figure 3). Participants who took part in FOBT had greater odds of meeting fruit and vegetable consumption guidelines across both time points, compared with non-participants (OR: 1.70, 95% CI: 1.14–2.55). There was no interaction between group and time for fruit and vegetable consumption (OR: 1.02, 95% CI: 0.66–1.58). FOBT participants and non-participants were equally likely to change their behaviour.
- *Physical activity*: The proportion of men taking part in MPA once or more per week did not change between baseline (88.6%) and follow-up (85.5%) measurements (n = 736; OR: 0.75, 95% CI: 0.49–1.15) (Figure 4). No differences in MPA were observed between FOBT participants and FOBT non-participants (OR: 1.08, 95% CI: 0.69–1.71). There was no group-by-time interaction (OR: 0.97, 95% CI: 0.57–1.67). The proportion of men taking part in VPA once or more per week did not change between baseline (40.1%) and follow-up (41.2%) measurements (n = 734; OR: 0.83, 95% CI: 0.64–1.01). There was no main effect of group on the proportion of men participating in VPA once or more per week (OR: 0.79, 95% CI: 0.57–1.08). A group-by-time interaction was found (OR: 1.40, 95% CI: 1.01–1.95) (Figure 4). Among men who participated in FOBT, the proportion taking part in VPA once or more per week increased over time (38.7% to 43.2%). For FOBT non-participants, the proportion taking part in VPA once or more per week decreased over time (41.6% to 37.5%).

## Discussion

In this cohort of men, we did not find evidence of spontaneous lifestyle changes following first FOBT participation for smoking, alcohol consumption, fruit and vegetable intake, nor MPA. A modest increase in VPA among FOBT attendees provides tentative support for screening participation as a teachable moment for PA, but effect sizes were small.

These results are in line with most studies in this area that have not observed spontaneous changes in behaviour following cancer screening participation.^10,11^ Our study adds more robust evidence that screening is unlikely to prompt improvements for most behaviours, as it used a prospective cohort design, with a sample that is more representative of the general population compared with previous research. We also controlled for retirement, which may be related to both levels of physical activity and FOBT uptake.^22,23^ The finding that VPA may increase after participating in colorectal cancer screening is similar to a previous study, which observed changes to PA following participation in a bowel scope trial.^3^ The same study also observed positive changes to smoking behaviour and fruit and vegetable consumption. Our study, which included a non-screened comparison group, found that smoking decreased among FOBT attendees and non-attendees. This suggests that although smoking appears to reduce over time, FOBT participation is unlikely to be the catalyst for change.

Previous research has found that MPA and VPA reduce with age within the ELSA cohort, and interventions to increase PA among adults are generally only modestly effective.^24,25^ It is therefore encouraging that, in our sample, screening attenders appeared to increase their VPA. This effect needs to be replicated in additional cohorts, although it is possible that FOBT screening may provide a teachable moment, prompting spontaneous change for this behaviour. MPA remained stable over time and did not decrease as VPA increased.^26^ However, almost 90% of the sample reported engaging in MPA once or more per week at baseline, suggesting a ceiling effect. Research exploring the mechanisms involved in creating teachable moments is sparse. It has been theorised that changes to a number of constructs including perceived risk, affect and self-concept may prompt behaviour change.^27^

Despite an absence of evidence to support positive changes to multiple health behaviours following FOBT screening, our research does not support the suggestion of a ‘health certificate effect’.^6,7^ It has been suggested that positive behaviour change following screening may be observed predominantly among people who receive abnormal screening outcomes.^10^ Although screening results were unknown for our participants, the exclusion of participants with a diagnosis of cancer means it is likely that the majority received a normal screening result. Only 2.5% of men will receive an abnormal result; therefore, studies with larger sample sizes are needed to determine whether FOBT result has an impact on behaviour change.^28^

In line with previous research,^29^ FOBT participants were less likely to smoke, and more likely to meet guidelines for fruit and vegetable consumption, compared with non-participants. Health behaviours have been found to cluster,^30^ and this may reflect greater health awareness among this group. However, although the FOBT group displayed healthier behaviours than the non-screened group, health behaviours were sub-optimal. The proportion of men consuming alcohol in excess of current guidelines increased from baseline to follow-up, suggesting there may be a need for interventions targeting this behaviour among men in this age group. In line with recent public health initiatives, such as Making Every Contact Count,^31^ cancer screening could provide an opportunity to deliver interventions.^1^

Previous, more intensive, behaviour change interventions in the screening context have predominantly targeted patients with screen-detected polyps; however, most people will receive a normal screening result. Trials of these interventions, aimed at promoting diet, PA and weight loss, have documented encouraging changes to behaviour.^32–34^ Trials are needed to test interventions delivered during different screening procedures, and the feasibility of delivering this kind of information to all screening attendees.

The ELSA is said to be broadly representative of British older adults.^16^ We compared our participants with 2011 Census data (limited to English men, aged 60–64) for two key demographic variables: ethnicity and education. The proportion of white participants (Census = 94.4%, ELSA = 95.1%) and participants with education to degree level or above (Census =25.2%, ELSA = 27.7%) were similar. While our sample appears to be broadly representative of men aged 60–64 for ethnicity and education, our findings may not be generalisable to other groups. It is important to determine if the findings observed in this study are similar for attendees of other screening programmes, for women, and for non-naïve attenders.

Within our sample, self-reported uptake of FOBT was 63%, compared with 54% uptake in the general population.^35^ Estimates of the accuracy of self-reported FOBT uptake vary, with some research suggesting it can be highly accurate, and others noting a 13% overestimation.^36,37^ Despite the ELSA being broadly representative for demographic factors, it is possible that certain health behaviours, such as screening participation, are over-represented. ELSA participants may be more health conscious, and therefore more likely to take part in cancer preventive behaviours, or taking part in the ELSA may have an impact on health behaviours. We do not have any information on reasons for FOBT non-participation. It is likely that these individuals chose not to participate, as opposed to missing an invitation, but this cannot be confirmed.

There are differences in how behaviour change is measured at cancer screening, with some research assessing change across a combined behavioural score.^6,38^ Our research examined the impact of screening on individual behaviours; however, the ELSA did not include measures of all behavioural risk factors for bowel cancer, such as the consumption of red and processed meat, with fruit and vegetable consumption the only measure of diet. The items used to measure VPA and MPA meant that it was impossible to determine if people were meeting guidelines, and made it difficult to accurately gauge changes in PA. National estimates of PA are usually based on the number of minutes of MPA and VPA completed per day or week.^39^ Research using objective measures of health behaviours is needed, to explore whether the changes observed in this study are reliable. Despite offering a different perspective from research conducted within screening trials, using a prospective cohort research design prevents confirmation of causality. Finally, this research design may fail to capture transient changes in health behaviours which might be made following cancer screening. Therefore, more research is needed to understand the timeliness of the teachable moment in the cancer screening context.

## Conclusion

FOBT participation did not appear to prompt long-standing, spontaneous, positive changes to multiple health behaviours within this sample of male ELSA participants, although modest spontaneous behaviour change was observed for VPA. FOBT participation did not appear to discourage behaviour change. Future research should investigate whether spontaneous lifestyle changes occur across other cancer screening programmes, the mechanisms involved in creating the teachable moment and the appetite for lifestyle advice in the cancer screening setting.

## References

[1] Senore C Giordano L Bellisario C , et al. Population based cancer screening programmes as a teachable moment for primary prevention interventions. A review of the literature. Front Oncol 2012; 2: 45–45. 22649789 10.3389/fonc.2012.00045 PMC3355877

[2] Lawson PJ Flocke SA. Teachable moments for health behavior change: a concept analysis. Patient Educ Couns 2009; 76: 25–30. 19110395 10.1016/j.pec.2008.11.002 PMC2733160

[3] Miles A Wardle J McCaffery K , et al. The effects of colorectal cancer screening on health attitudes and practices. Cancer Epidemiol Biomarkers Prev 2003; 12: 651–655. 12869406

[4] Stewart-Brown S Farmer A. Screening could seriously damage your health. BMJ 1997; 314: 533–534. 9055702 10.1136/bmj.314.7080.533 PMC2126029

[5] Tymstra T Bieleman B. The psychosocial impact of mass-screening for cardiovascular risk-factors. Fam Pract 1987; 4: 287–290. 3692036 10.1093/fampra/4.4.287

[6] Berstad P Loberg M Larsen IK , et al. Long-term lifestyle changes after colorectal cancer screening: randomised controlled trial. Gut 2015; 64: 1268–1276. 25183203 10.1136/gutjnl-2014-307376

[7] Larsen IK Grotmol T Almendingen K , et al. Impact of colorectal cancer screening on future lifestyle choices: a three-year randomized controlled trial. Clin Gastroenterol Hepatol 2007; 5: 477–483. 17363335 10.1016/j.cgh.2006.12.011

[8] Hoff G Thiis-Evensen E Grotmol T , et al. Do undesirable effects of screening affect all-cause mortality in flexible sigmoidoscopy programmes? Experience from the Telemark Polyp Study 1983–1996. Eur J Cancer Prev 2001; 10: 131–137. 11330453 10.1097/00008469-200104000-00003

[9] Cooper GC Harvie MN French DP. Do negative screening test results cause false reassurance? A systematic review. Br J Health Psychol 2017; 22: 958–977. 28895257 10.1111/bjhp.12265

[10] Slatore CG Baumann C Pappas M , et al. Smoking behaviors among patients receiving computed tomography for lung cancer screening. Systematic review in support of the U.S. preventive services task force. Annals Am Thorac Soc 2014; 11: 619–627. 10.1513/AnnalsATS.201312-460OC 24701999

[11] van der Aalst CM van Klaveren RJ de Koning HJ. Does participation to screening unintentionally influence lifestyle behaviour and thus lifestyle-related morbidity? Best Pract Res Clin Gastroenterol 2010; 24: 465–478. 20833350 10.1016/j.bpg.2010.06.001

[12] Bankhead CR Brett J Bukach C , et al. The impact of screening on future health-promoting behaviours and health beliefs: a systematic review. Health Technol Assess 2003; 7: 1–92. 10.3310/hta7420 14670217

[13] van der Aalst CM van den Bergh KA Willemsen MC , et al. Lung cancer screening and smoking abstinence: 2 year follow-up data from the Dutch-Belgian randomised controlled lung cancer screening trial. Thorax 2010; 65: 600–605. 20627916 10.1136/thx.2009.133751

[14] Bretteville-Jensen AL Biorn E Selmer R. Does screening participation affect cigarette smokers' decision to quit? A long-horizon panel data analysis. Nordic Stud Alcohol Drugs 2014; 31: 141–160.

[15] Department of Health. Improving outcomes: a strategy for cancer. London: Author, 2011.

[16] Steptoe A Breeze E Banks J , et al. Cohort profile: the English longitudinal study of ageing. Int J Epidemiol 2013; 42: 1640–1648. 23143611 10.1093/ije/dys168 PMC3900867

[17] Department of Health. *UK Chief Medical Officers’ Alcohol guidelines review: summary of the proposed new guidelines*, 2016. https://assets.publishing.service.gov.uk/government/uploads/system/uploads/attachment_data/file/489795/summary.pdf

[18] Public Health England. *A quick guide to the government’s healthy eating recommendations*, 2017. https://assets.publishing.service.gov.uk/government/uploads/system/uploads/attachment_data/file/742746/A_quick_guide_to_govt_healthy_eating_update.pdf

[19] Department of Health. *Start active, stay active: a report on physical activity for health from the four home countries’ Chief Medical Officers*, 2011. https://assets.publishing.service.gov.uk/government/uploads/system/uploads/attachment_data/file/216370/dh_128210.pdf

[20] Hanley JA Negassa A Edwardes MDD , et al. Statistical analysis of correlated data using generalized estimating equations: an orientation. Am J Epidemiol 2003; 157: 364–375. 12578807 10.1093/aje/kwf215

[21] Williams K Steptoe A Wardle J. Is a cancer diagnosis a trigger for health behaviour change? Findings from a prospective, population-based study. Br J Cancer 2013; 108: 2407–2412. 23695026 10.1038/bjc.2013.254 PMC3681023

[22] Kelly S Olanrewaju O Cowan A , et al. Interventions to prevent and reduce excessive alcohol consumption in older people: a systematic review and meta-analysis. Age Ageing 2018; 47: 175–184. 28985250 10.1093/ageing/afx132 PMC6016606

[23] Weber MF Cunich M Smith DP , et al. Sociodemographic and health-related predictors of self-reported mammogram, faecal occult blood test and prostate specific antigen test use in a large Australian study. BMC Public Health 2013; 13: 429. 23641775 10.1186/1471-2458-13-429 PMC3663683

[24] Smith L Gardner B Fisher A , et al. Patterns and correlates of physical activity behaviour over 10 years in older adults: prospective analyses from the English Longitudinal Study of Ageing. BMJ Open 2015; 5: e007423. 10.1136/bmjopen-2014-007423 PMC4401868 25877281

[25] Conn VS Hafdahl AR Mehr DR. Interventions to increase physical activity among healthy adults: meta-analysis of outcomes. Am J Public Health 2011; 101: 751–758. 21330590 10.2105/AJPH.2010.194381 PMC3052337

[26] Gomersall SR Rowlands AV English C , et al. The ActivityStat hypothesis the concept, the evidence and the methodologies. Sports Med 2013; 43: 135–149. 23329607 10.1007/s40279-012-0008-7

[27] McBride CM Ostroff JS. Teachable moments for promoting smoking cessation: the context of cancer care and survivorship. Cancer Control 2003; 10: 325–333. 12915811 10.1177/107327480301000407

[28] Logan RF Patnick J Nickerson C , et al. Outcomes of the Bowel Cancer Screening Programme (BCSP) in England after the first 1 million tests. Gut 2012; 61: 1439–1446. 22156981 10.1136/gutjnl-2011-300843 PMC3437782

[29] Shapiro JA Seeff LC Nadel MR. Colorectal cancer-screening tests and associated health behaviors. Am J Prev Med 2001; 21: 132–137. 11457633 10.1016/s0749-3797(01)00329-4

[30] Mawditt C Sacker A Britton A , et al. The clustering of health-related behaviours in a British population sample: testing for cohort differences. Prev Med 2016; 88: 95–107. 27058943 10.1016/j.ypmed.2016.03.003

[31] Public Health England. *Making Every Contact Count (MECC): Consensus statement*, 2016. https://www.england.nhs.uk/wp-content/uploads/2016/04/making-every-contact-count.pdf

[32] Caswell S Anderson AS Steele RJ. Bowel health to better health: a minimal contact lifestyle intervention for people at increased risk of colorectal cancer. Br J Nutr 2009; 102: 1541–1546. 19640325 10.1017/S0007114509990808

[33] Anderson AS Caswell S Macleod M , et al. Awareness of lifestyle and colorectal cancer risk: findings from the BeWEL study. Biomed Res Int 2015; 2015: 871613. 26504842 10.1155/2015/871613 PMC4609381

[34] Baker AH Wardle J. Increasing fruit and vegetable intake among adults attending colorectal cancer screening: the efficacy of a brief tailored intervention. Cancer Epidemiol Biomarkers Prev 2002; 11: 203–206. 11867508

[35] von Wagner C Baio G Raine R , et al. Inequalities in participation in an organized national colorectal cancer screening programme: results from the first 2.6 million invitations in England. Int J Epidemiol 2011; 40: 712–718. 21330344 10.1093/ije/dyr008

[36] Lo SH Waller J Vrinten C , et al. Self-reported and objectively recorded colorectal cancer screening participation in England. J Med Screen 2016; 23: 17–23. 26408533 10.1177/0969141315599015 PMC4741296

[37] Jones RM Mongin SJ Lazovich D , et al. Validity of four self-reported colorectal cancer screening modalities in a general population: differences over time and by intervention assignment. Cancer Epidemiol Biomarkers Prev 2008; 17: 777–784. 18381476 10.1158/1055-9965.EPI-07-0441

[38] Helander S Heinavaara S Sarkeala T , et al. Lifestyle in population-based colorectal cancer screening over 2-year follow-up. Eur J Public Health 2018; 28: 333–338. 29020299 10.1093/eurpub/ckx139

[39] NHS Digital. *Health Survey for England 2016*, 2017. https://digital.nhs.uk/data-and-information/publications/statistical/health-survey-for-england/health-survey-for-england-2016

